# A Synthetic Interaction between *CDC20* and *RAD4* in *Saccharomyces cerevisiae* upon UV Irradiation

**DOI:** 10.1155/2014/519290

**Published:** 2014-02-23

**Authors:** Bernadette Connors, Lauren Rochelle, Asela Roberts, Graham Howard

**Affiliations:** ^1^Department of Biology, Dominican College, 470 Western Highway, Orangeburg, NY 10962, USA; ^2^Department of Cell Biology, Duke University, Durham, NC, USA; ^3^Millsaps College Jackson, MS, USA; ^4^ADAMS, Rome, GA, USA

## Abstract

Regulation of DNA repair can be achieved through ubiquitin-mediated degradation of transiently induced proteins. In *Saccharomyces cerevisiae*, Rad4 is involved in damage recognition during nucleotide excision repair (NER) and, in conjunction with Rad23, recruits other proteins to the site of damage. We identified a synthetic interaction upon UV exposure between Rad4 and Cdc20, a protein that modulates the activity of the anaphase promoting complex (APC/C), a multisubunit E3 ubiquitin ligase complex. The moderately UV sensitive Δ*rad4* strain became highly sensitive when *cdc20-1* was present, and was rescued by overexpression of *CDC20*. The double mutant is also deficient in elicting *RNR3-lacZ* transcription upon exposure to UV irradiation or 4-NQO compared with the Δ*rad4* single mutant. We demonstrate that the Δ*rad4*/*cdc20-1* double mutant is defective in double strand break repair by way of a plasmid end-joining assay, indicating that Rad4 acts to ensure that damaged DNA is repaired via a Cdc20-mediated mechanism. This study is the first to present evidence that Cdc20 may play a role in the degradation of proteins involved in nucleotide excision repair.

## 1. Introduction

Increased expression of genes necessary for detecting and repairing DNA damage can result when cells are exposed to certain genotoxic compounds [1–3]. These same treatments can also induce differential expression of genes in the ubiquitin- (Ub-) mediated proteolytic pathway [4, 5], suggesting interplay between DNA repair and protein degradation [6–9]. Mechanisms to transiently stabilize or reduce the abundance of repair proteins following detection and/or removal of DNA damage could provide a means for regulating these processes. Defects in Ub-mediated protein degradation have been linked to breast cancer [10], Angelman syndrome [11], von Hippel-Lindau disease [12], and altered responses to clinical anesthetics [13], while defects in DNA damage repair are associated with human disorders such as xeroderma pigmentosum, Cockayne syndrome, and trichothiodystrophy [14]. This faithful transmission of genetic material is critical to cell survival, while proper functioning of DNA repair processes ensures that genomic integrity is maintained.

Ubiquitination results in the modification of protein function, thereby controlling cellular processes such as cell cycle progression, differentiation, and stress responses [15, 16]. This 8.5 kDa protein is first activated by ubiquitin-activating enzymes (E1) before being transferred to ubiquitin-conjugating enzymes (E2) and ubiquitin protein ligases (E3) [17]. It is ultimately attached to lysine residues in target proteins, and multiubiquitin chains form through the activity of ubiquitin chain assembly factors (E4). These ubiquitinated proteins are then targeted to the 26S proteasome for degradation. The anaphase promoting complex/cyclosome (APC/C) is one of several multisubunit E3 ubiquitin ligases that control progression through the cell cycle [18, 19]. In addition to E1 and E2 enzymes, APC/C-mediated ubiquitination depends on the activator proteins, Cdc20 or Cdh1 [20–22], with the former regulating the metaphase to anaphase transition through the degradation of securin. Not surprisingly, APC/C^Cdc20^ is a target of the spindle checkpoint, not allowing the degradation of securin until proper attachment and alignment of all kinetochores to the spindle are completed [23]. Clarke et al. [24] have reported that in budding yeast Cdc20 is capable of acting independently of the APC/C, suggesting an alternative mechanism to its ability to regulate mitotic exit. In metazoans, the APC/C is also active in postmitotic differentiated cells, implying that it has assumed functions in nonproliferating cells as well [25].

Nucleotide excision repair (NER) is a highly conserved mechanism that detects and removes bulky lesions from DNA following chemical treatment or UV irradiation [26–28]. Compromising NER activity has pleiotropic effects, leading to increased mutation frequency and risk of carcinogenesis [29, 30]. Bulky adducts repaired by the NER include *N*-acetyl-2-aminofluorene (AAF) adducts, cyclobutane pyrimidine dimers (CPDs), (6-4) photoproducts, and cisplatin intrastrand crosslinks [31–33]. Global genome NER (GGR), responsible for the repair of untranscribed regions of the genome, can be divided into five distinct steps: damage recognition, incision, excision, repair synthesis, and DNA ligation [26]. The expression of a number of NER proteins is upregulated as a result of DNA damage; however, once the DNA damage is repaired and the proteins are no longer needed, they are degraded to restore basal levels [1, 34]. Multiple interactions between members of the NER, ubiquitin-associated enzymes, and proteasomal subunits have been revealed by both genetic and biochemical approaches [5–7, 9, 35, 36]. This would indicate that one means of regulating the repair process is through Ub-mediated degradation of NER proteins once they are no longer needed.

Many of the more than 30 NER proteins are essential to the repair processes; however, a subset is considered accessory, and deletion of these results in a less severe sensitivity to DNA damaging agents in comparison to the essential components, which have near 0% survival with UV treatment [37–39]. Rad4 and Rad23 are two such accessory members, interacting strongly with one another to control the damage recognition step of NER [40, 41]. Rad4, which transiently accumulates following UV irradiation [42], is stabilized by Rad23 and this interaction stimulates binding of the Rad4/Rad23 complex to the damaged DNA [5, 43]. Ng et al. [8] have concluded that the primary function of Rad23 in NER is the stabilization of Rad4, although diminished Rad4 stability is not the primary cause of deficient NER in *rad23* mutant cells [9, 44].

To examine the interplay between Cdc20 and NER, we utilized yeast strains deleted for *RAD4* and harboring the conditional *cdc20-1* mutation. We speculate that Cdc20 functions to modulate components of the NER. We report here that the diminished capacity of Cdc20 results in extreme UV sensitivity in NER-compromised mutants of *S. cerevisiae*, specifically those harboring Δ*rad4*.

## 2. Materials and Methods

### 2.1. Strain Construction

All deletion strains used in this study carry a gene deletion linked to a kanamycin-resistance marker *kanMX *that confers resistance to the antibiotic Geneticin (G418) and were obtained from the Mississippi Functional Genomic Network (MFGN) core facility (University of Southern Mississippi, Hattiesburg, MS) or Open Biosystems (now Thermo Fisher Scientific). A *cdc20-1* mutant strain containing the CloNAT resistance marker was generated using a one-step gene replacement. Double mutants harboring various gene deletions and *cdc20-1* were also generated by one-step gene replacement, making the single and double mutants isogenic with respect to one another. All other double mutants were generated by traditional crosses and verified by temperature sensitivity and dual resistance to G418 and CloNAT.

### 2.2. Plasmid Constructions

A plasmid containing *cdc20*-*1* was constructed by amplifying the mutant allele from strain 405-1-1 (gift from D. Burke), which included 400 bp upstream of the start codon and 500 bp downstream of the stop codon. The amplicon was then ligated into pGEM-T (Promega). The CloNAT resistance gene was excised from p4339 (obtained from MFGN) and blunted. The fragment was ligated at a *Stu*I site 150 bp downstream of the stop codon of *cdc20-1*. DNA sequencing was performed to verify the presence of the *cdc20-1* mutation and the resistance marker. The resulting recombinant plasmid was linearized with *Spe*I to be used for one-step gene replacement.

Plasmids designed to overexpress *CDC20 *were generated using the pYES.2 vector (Invitrogen), which carries a *URA3* marker, 2 *μ* ori, and multiple cloning site downstream of the *gal1* inducible promoter. Plasmids harboring *CDC20* or *cdc20-1* were constructed by ligating a 2.0 kb *Hind*III fragment into pYES.2 at the *Hind*III site and orientation was verified through restriction enzyme analysis. All other overexpression plasmids were obtained from Open Biosystems, in which the genes of interest were subcloned behind the *gal1* inducible promoter in the BG1805 plasmid vector. All yeast transformations were performed with the high efficiency LiOAc method [45].

### 2.3. Drug Cytotoxicity Assays

Yeast strains were incubated at 22°C for 12–16 hours in yeast-peptone-dextrose (YPD) or the appropriate selective medium. Cultures were normalized to an *A*_660_ of 1.0, and a 1 : 10 serial dilution was performed in sterile water. From each dilution, 5 *μ*L was spotted onto YPD agar containing methyl methanesulfonate (MMS) (0.05%), hydroxyurea (100 mM), phleomycin (5 *μ*g/mL), benomyl (12 *μ*g/mL), and 4-nitroquinoline (4-NQO) (0.2, 0.5, and 1.0 *μ*g/mL) or exposed to 10 or 25 J/m^2^ of UV radiation in a Stratalinker model 1800 (Stratagene). For those strains harboring genes to be overexpressed, transformants were assayed on synthetic complete dextrose media lacking uracil and containing either 2% glucose or 3% galactose/0.5% glucose.

Quantification of survival was determined by plating a known number of cells on selective media, followed by UV irradiation (10 and 25 J/m^2^) and incubation at 22°C for 3-4 days. UV survival was calculated by dividing the number of surviving colonies following treatment by the number of colonies on untreated plates. Three independent trials were conducted for each treatment.

### 2.4. *β*-Galactosidase Assays

The *β*-gal activity assay was performed as described previously [46]. Briefly, 0.5 mL of an overnight culture was used to inoculate 4.5 mL of fresh selective media. After 8–10 hours at 22°C, cells were treated with 0.2, 0.5, or 1.0 *μ*g/mL of 4-NQO or UV irradiated at 10, 25, or 50 J/m^2^. Cells were returned to 22°C for another 4 hours prior to performing the *β*-gal assay. Four hours was found to be optimal for induction of the RNR3:*lacZ* fusion protein in studies by Jia and Xiao [46]. Activities were determined, and fold induction was calculated as a ratio of *β*-galactosidase activity in the cultures with and without treatment.

### 2.5. Plasmid End Joining Assay

The pYES.2 vector (Invitrogen) was digested with *Pvu*II, cutting the plasmid outside of the* URA3* marker. Linearized and uncut plasmids of a known concentration were transformed in parallel into multiple strains and plated on SCD lacking uracil. Following 3-4 days of incubation at 22°C to allow for sufficient colony growth, colonies were counted. Transformation efficiencies were calculated using the Transformation Efficiency Calculator (http://www.sciencegateway.org/tools/transform.htm). The results are the average of at least three independent experiments, and standard error of the mean was determined.

### 2.6. Microscopic Analyses

A strain carrying *RAD4* with a C-terminal GFP fusion was obtained from the MFGN core facility. The *cdc20*-*1 *allele was introduced into this strain via a traditional cross, and gene replacement was verified by PCR, resistance to CloNAT, and temperature sensitivity at 37°C. Cultures were grown at 22°C in SCD lacking histidine with or without CloNAT for 12–16 hours and subsequently UV irradiated at 25 J/m^2^ and 100 J/m^2^. An outgrowth of 4 hours was allowed before cells were briefly centrifuged and washed once with water. This outgrowth time was chosen as Lommel et al. [5] have shown the half-life of Rad4 to be 4 hours. Samples were examined and photographed on a Zeiss Imager M1 AXIO fluorescence microscope paired with a Photometrics Coolsnap HQ^2^camera. UV exposures were done in triplicate, and greater than 150 cells were counted and analyzed for each treatment.

## 3. Results

### 3.1. Mutation in *CDC20* Results in Increased Sensitivity to Ultraviolet Radiation in Δ*rad4* Backgrounds

**Figure 1:**
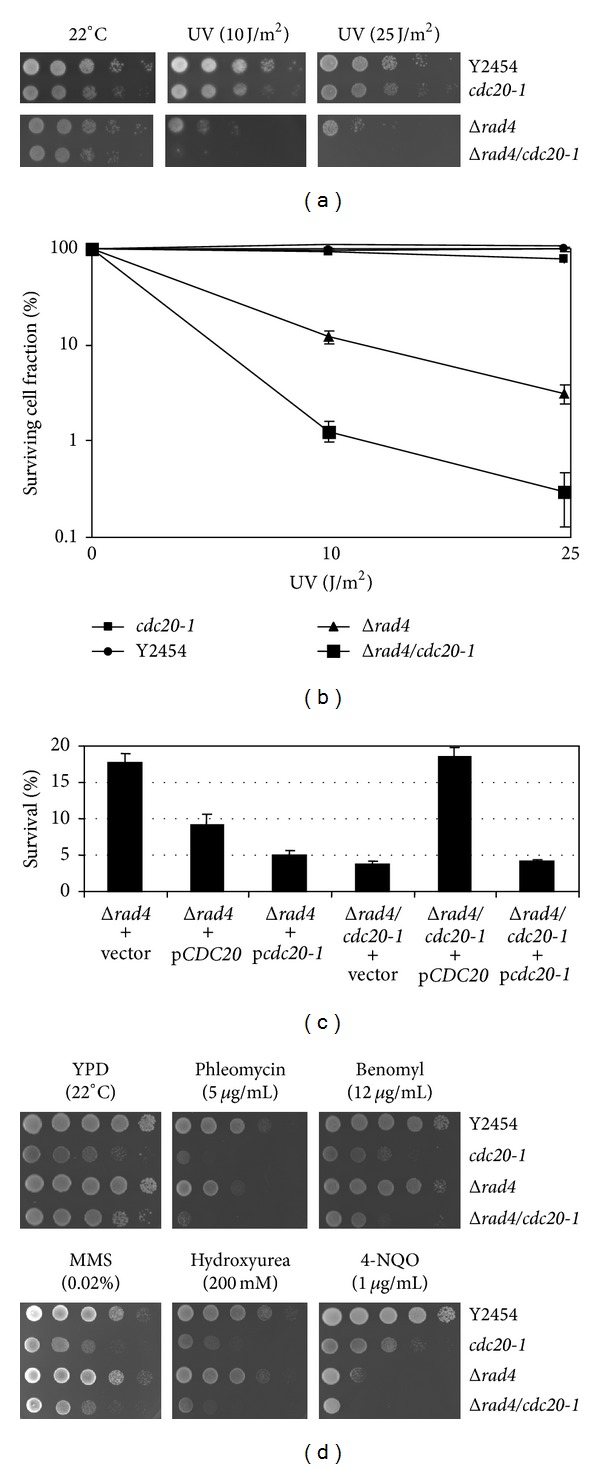
UV resistance of Δ*rad4 *mutants was diminished by the introduction of temperature-sensitive *cdc20-1* allele. (a) The optical density *A*_600_ of overnight cultures was normalized to 1.0, and a 1 : 10 serial dilution was prepared. Onto YPD, 5 *μ*L of each dilution was spotted and plates were exposed to UV radiation at energy levels corresponding to 10 and 25 J/m^2^. Plates were then incubated at 22°C for 3 days. The growth of Δ*rad4* harboring *cdc20-1* was greatly reduced relative to the Δ*rad4* single mutant or the wild-type strain (Y2454). (b) Diluted cultures of yeast were plated on YPD and then exposed to UV at energy levels of 10 and 25 J/m^2^, or none at all. Following 3 days of incubation at 22°C, colonies were counted and percent survival was calculated as the total number of colonies on the treated plates divided by the number of colonies on the untreated plates. The Δ*rad4/cdc20-1* mutant exhibits a tenfold decrease in survivability relative to the Δ*rad4* strain at both energy levels tested. (c) Plasmids were constructed in which either *CDC20* or *cdc20-1* was placed under the control of the *gal1* promoter. These plasmids, in addition to the empty vector, were transformed into yeast strains containing Δ*rad4* and Δ*rad4*/*cdc20-1*. Survivability was determined as described in (c), with cells plated on SCD lacking uracil rather than YPD. Overexpression of *CDC20* (pCDC20) resulted in increased survivability of the Δ*rad4*/*cdc20-1* strain, while overexpression of *cdc20-1 (pcdc20-1) *did not. (d) Yeast cells were prepared as in (a) and spotted onto YPD media containing 5 *μ*g/mL phleomycin, 12 *μ*g/mL benomyl, 0.02% MMS, 200 mM hydroxyurea, and 1 *μ*g/mL 4-NQO. The Δ*rad4*/*cdc20-1* strain exhibited decreased growth on 4-NQO relative to Δ*rad4* but was otherwise similar in growth patterns to the *cdc20-1 *mutant.

The Δ*rad4*/*cdc20-1 *double mutant was initially identified as displaying a synthetic sick phenotype in a synthetic genome analysis using a query strain harboring the *cdc20-1* temperature sensitive allele (Figure 1(a)). The UV sensitivity of the Δ*rad4* strain was increased approximately 10-fold by the point mutation in *cdc20-1 *(Figure 1(b)). When *CDC20 *under the control of the *gal1* promoter was reintroduced into the double mutant strain and then irradiated, UV resistance comparable to that seen in Δ*rad4 *strains was restored (Figure 1(c)). This was not observed when *cdc20-1 *was transformed into the double mutant. When *cdc20-1* was introduced into the Δ*rad4 *strain survival at 10 J/m^2^was less than that caused when *CDC20* was overexpressed in the same strain, indicating a dominant negative phenotype of the *cdc20-1* mutant allele. The sensitivities of neither the single nor double mutant to hydroxyurea, MMS, phleomycin, and benomyl were significantly different as compared to wild-type or the *cdc20-1* strains alone (Figure 1(d)).

### 3.2. Δ*rad4* Enhances *RNR3*:*lacZ* Induction While Δ*rad4*/*cdc20-1 *Compromises Induction upon Exposure to UV Irradiation and 4-NQO

**Figure 2:**
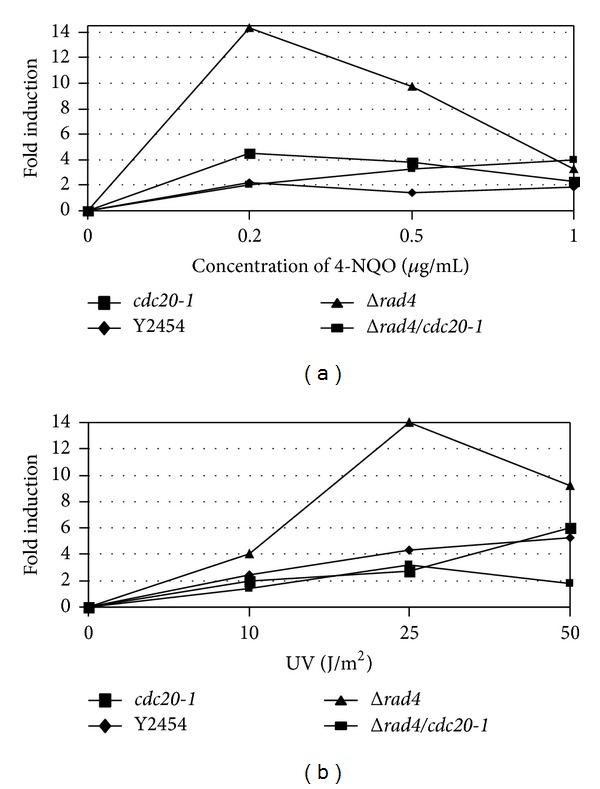
DNA damage-induced *RNR3*-*lacZ* expression is reduced in Δ*rad4/cdc20 *mutants that have been (a) exposed to 4-NQO or (b) UV irradiated. Strains were transformed with pZZ2 (gift from S. Elledge), which contains the large subunit of the ribonucleotide reductase complex (*RNR3*) fused in-frame to *lacZ. *Cultures were exposed to UV or 4-NQO, incubated for 4 hours, and assayed for *β*-galactosidase activity. Fold induction was calculated as the ratio of *β*-gal activity of treated cultures cells to that of untreated cultures. In both treatments, Δ*rad4* strains were capable of evoking *RNR3*-*lacZ* expression, while Δ*rad4/cdc20-1 *strains did not significantly affect this activity relative to wild-type (Y2454) or *cdc20-1* strains.

It has been reported that inactivation of certain DNA repair pathways in *S. cerevisiae* may diminish or enhance gene expression in response to DNA damage [46]. In this assay, plasmid with *RNR3* fused to *lacZ* was transformed into *Δrad4*/*cdc20-1, *Δ*rad4, cdc20-1*, and wild-type strains. RNR3 is the large subunit of the ribonucleotide reductase complex, which catalyzes dNTP synthesis and is subject to both DNA replication and repair checkpoints. We examined the response of Δ*rad4* and Δ*rad4/cdc20-1 *strains to both UV radiation and 4-NQO. Deletion of *RAD4* enhanced *RNR3*-*lacZ* induction by 4-NQO 3.5- and 2.5-fold at concentrations of 0.2 and 0.5 *μ*g/mL (Figure 2(a)). UV radiation at energy levels of 10 and 25 J/m^2^ enhanced this induction by 2- and 7-fold, respectively (Figure 2(b)). With the introduction of *cdc20-1 *into the Δ*rad4 *background, however, the fold increase was diminished and not different than either wild-type or *cdc20-1* strains alone. These data are not consistent with the values for survivability following UV exposure and indicate an altered capacity for DNA repair that is not related to cell survival in strains harboring both Δ*rad4 *and *cdc20-1 *simultaneously.

### 3.3. Δ*rad4*/*cdc20-1* Mutant Strains Are Defective in Plasmid Rejoining *In Vivo*

**Figure 3:**
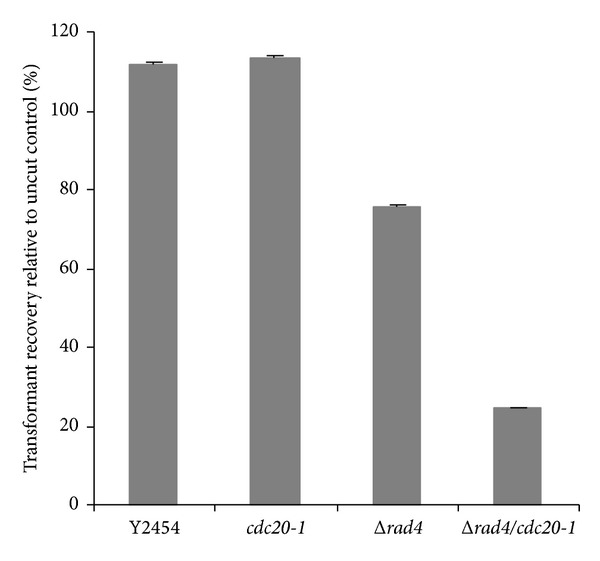
Activity of Cdc20 contributes to the ability of Δ*rad4 *strains to carry out double strand break repair. Plasmid DNA (pYES.2/*URA3* ^+^) was linearized with *PvuII, *and aliquots from the same pool of digested DNA were used to transform Δ*rad4*, Δ*rad4*/*cdc20-1*, *cdc20-1*, or a wild-type strain (Y2454). Following incubation at 22°C for 4 days, colonies were counted, and transformation efficiencies were calculated. The fold changes (linearized/uncut) are represented.

To determine whether introduction of *cdc20-1* affected the cell's ability to repair double strand breaks, a plasmid end-joining assay was performed [47, 48]. Briefly, strains of *S. cerevisiae *were transformed with a linearized plasmid (pYES.2) containing the *URA3* marker. To normalize for differences in transformation efficiencies between strains, an uncut version of the same plasmid was transformed, in parallel. Since the plasmid must be recircularized in order to revert the *ura* ^−^ phenotype of the selected strains, the number of transformants obtained with the linearized plasmid normalized to the number obtained with the uncut plasmid provides a quantitation of the DSB-repair ability of the yeast strains. As shown in Figure 3, Δ*rad4* strains were able to repair DSBs, although not as efficiently as wild type. The double mutant, however, had significantly diminished ability to repair these defects as compared with the single deletion alone. These results indicate that introduction of *cdc20-1* affects the cells ability to repair damaged DNA in an NER-defective strain.

### 3.4. Microscopic Analysis of *Rad4-GFP*/*cdc20-1* Mutants Reveals Altered Rad4 Fluorescence upon UV Irradiation

**Figure 4:**
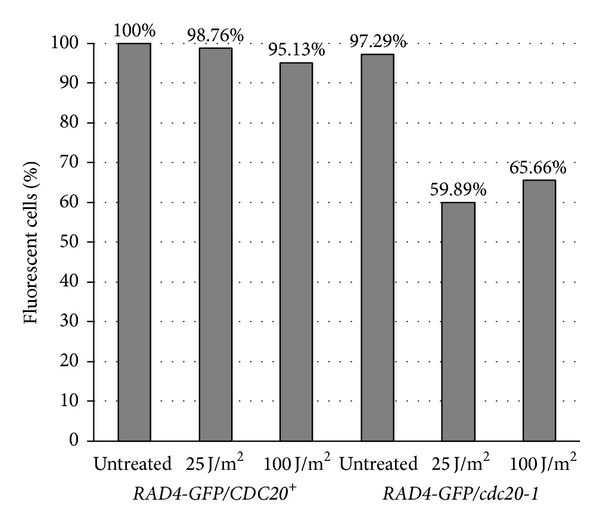
The *cdc20-1 *mutant allele contributes to diminished expression of Rad4-GFP upon UV irradiation. Strains harboring *cdc20-1* show less fluorescence of Rad4-GFP. Cultures were grown overnight at 22°C and then UV irradiated using a Stratagene UV Stratalinker 1800. An outgrowth of four hours was then allowed. Three replicates were performed, and percentages were determined on a sample size of greater than 150 cells. All images were captured using a Zeiss Imager M1 AXIO paired with a Photometrics Coolsnap HQ^2^camera.

To determine whether *cdc20-1 *may have an effect on expression or stability of *RAD4* we utilized a strain of yeast harboring a C-terminal GFP tag on *RAD4*, with or without a chromosomal copy of *cdc20-1.* Liquid cultures were irradiated at 25 and 100 J/m^2^ and examined four hours after exposure. Fluorescence was observed in strains carrying the tagged copy of *RAD4* in an otherwise wild-type background, regardless of treatment or time of outgrowth. When *cdc20-1 *was present, however, fluorescence was diminished following UV irradiation such that around 60% of the cells had visible fluorescence (Figure 4). Since strains with *cdc20-1* were capable of survival on solid media at these energy levels, we do not attribute decreased fluorescence to cell death.

## 4. Discussion

Here we demonstrate that Δ*rad4* and *cdc20-1* exhibit a synthetic growth defect upon UV exposure in *Saccharomyces cerevisiae*. We examined strains carrying a deletion of *RAD4* and the conditional *cdc20-1* allele and found that the two mutations in combination resulted in extreme UV sensitivity. Overexpression of *CDC20 *in Δ*rad4*/*cdc20-1* restored the slight UV resistance observed in the single mutant Δ*rad4*. Microscopic analyses indicate that in the presence of *cdc20-1*, Rad4-GFP fluorescence is significantly diminished relative to wild type after a four outgrowth following UV irradiation. When introduced into a NER-defective strain (Δ*rad4*), survival following UV irradiation is severely compromised. Our data demonstrate that this UV sensitivity may be the result of a reduced capability to repair double strand breaks, as the Δ*rad4/cdc20-1* strain had a diminished capacity to anneal DSBs in a plasmid end-joining assay.

We propose that Cdc20 may act to indirectly modulate the nucleotide excision repair pathway by way of its role in protein degradation. In a report by Gillette et al. [9] a novel cullin-based E3 ubiquitin ligase (ECS ligase) was identified that ubiquitinates Rad4 following UV exposure. If Cdc20 has a role in regulation of ECS ligase activity, the ubiquitination of Rad4 could be affected indirectly; however, Cdc20 may also directly regulate its ubiquitination through the APC/C. Rad4 contains a putative cyclin destruction box (D box) in its amino terminus, although its expression does not exhibit cell cycle periodicity. D box motifs contain two conserved residues (RXXL) and several other more moderately conserved residues. They are a highly conserved sequence common to substrates of the APC/C [49–52]. Previous studies have shown that Rad4 is stable, with a half-life of approximately 4 hours [5]. Upon UV exposure, these levels decrease as the protein is proteolyzed by the 26S-proteasome. The ubiquitination, not the stabilization, of Rad4 in response to UV irradiation via the ECS ligase regulates NER in part [9]. Without an examination of Rad4 ubiquitination in *cdc20-1* mutants, however, it is impossible for us to state that this is indeed the case, although microscopic examinations lend proof to this argument.

Despite the strong genetic evidence that Cdc20 affects the ability of *Saccharomyces cerevisiae* to carry out NER in the absence of *RAD4* we have yet to demonstrate biochemically how Cdc20 acts in this pathway. There are a number of NER proteins that contain putative D boxes and many other candidate proteins that may be targeted for ubiquitination via the APC/C^Cdc20^ or acted upon by Cdc20 alone. We do not exclude the possibility that unidentified protein is a member of the chromatin remodeling complex as Rad4/Rad23 complex is known to have a role in maintenance of heterochromatin structure [53, 54]. In addition, recent studies have shown that Rad4 can bind to the proteasome without affecting overall protein degradation ability of the complex [36], and studies have yet to elucidate mediators of this direct binding. Further research is necessary to dissect this interaction and to understand the roles of each component. This study is the first, however, to present evidence that Cdc20 may play a role in the degradation of proteins involved in nucleotide excision repair.
